# Organisational management of hospital blood transfusion services in Nairobi County, Kenya: Evidence of implementation

**DOI:** 10.4102/ajlm.v8i1.676

**Published:** 2019-08-26

**Authors:** Anne N. Mbuthia, Eunice M. Mwangi, Musa O. Ong’ombe

**Affiliations:** 1 Kenya Methodist University, Nairobi, Kenya

**Keywords:** blood transfusion, quality system, organisational management, quality management system

## Abstract

**Background:**

The World Health Organization in 2002 recommended implementation of a quality system for national blood programmes to ensure adequate and safe blood products to patients. Key elements of the quality system include organisational management, standards, documentation, training and assessment.

**Objectives:**

The aim of this study was to describe the extent to which organisational management, which is the first element of a quality system, has been implemented in hospitals in Nairobi County, Kenya.

**Methods:**

A descriptive, cross-sectional study design was used. Sixty health workers were selected as respondents from 15 hospitals that provide blood transfusion services in Nairobi County. The data collection period was from June to August 2015 and the data were analysed in 2016.

**Results:**

Faith-based hospitals had the lowest level of organisational management implementation (33.3%), private hospitals had 42.5%, whereas government hospitals had the highest implementation (60%). The extent of implementation was based on performance of the senior management team, overall rated by the respondents at 40.1%, establishment of hospital transfusion committees in nine (60%) of the hospitals and appointment of key staff – quality officers in three (20%) hospitals and blood transfusion specialists in six (40%) hospitals. These key staff were instrumental in steering the quality system and ensuring sound blood transfusion practices.

**Conclusion:**

The implementation of quality management systems in hospital blood transfusion services can be improved through commitment from senior management teams, who should provide the necessary resources for employment of key staff and establish and empower hospital transfusion committees to guide the blood transfusion services.

## Introduction

Blood transfusion services are a key life-saving function in every health system. Scarcity of blood poses serious challenges, which sometimes hinder the ability to improve health outcomes.^1,2^ This is further compounded by wastage, inappropriate utilisation or storage and failure to maintain the cold chain. Availability of safe blood is a recurrent challenge in hospital blood transfusion services in Kenya. This is because of high demand for blood, especially for emergency obstetric care.^3^ The issue of scarcity is further compounded by challenges such as poor documentation, inadequate knowledge regarding the use of blood components and lack of haemovigilance tools.^4^ These shortcomings negatively affect the overall quality of service and contribute to wastage and inappropriate usage.

Comprehensive quality management systems serve two main purposes within blood transfusion services. Firstly, they ensure the services and blood components are safe, adequate and effective.^2^ Secondly, they provide data on adverse events for the national haemovigilance system.^1^ To achieve this, a robust quality management system is necessary at individual hospital blood transfusion services. To improve blood transfusion services, the World Health Organization (WHO) recommends adoption of a quality system that has five key elements: organisational management, implementation of quality standards, documentation, training of staff and regular assessment.^5^

There are three key areas of organisational management in blood transfusion services: a well-defined organisational structure that describes obligation, authority and responsibility, good planning for quality and consistency in service delivery, and leaders perfecting a culture that integrates quality into all activities.^6^ The senior management team ensures quality and consistency in service delivery by establishing a multidisciplinary hospital blood transfusion committee, appointing quality officers and blood transfusion specialists who are key members of the transfusion committee and providing resources to run the service. In Kenya, the responsibilities of a quality officer include haemovigilance to monitor trends of adverse reactions and hospital response, track blood utilisation, evaluate hospital transfusion practice, and prepare monthly reports for the transfusion committee and regional blood transfusion centre (RBTC).^7^ Blood transfusion specialists support blood management best practices, which eliminate, reduce or optimise blood transfusions to improve health outcomes.^8^ Other key personnel include the blood bank manager who directs and controls operations, and the operational staff (laboratory technologists, nurses and clinicians) who offer direct services to patients.

**FIGURE 1:**
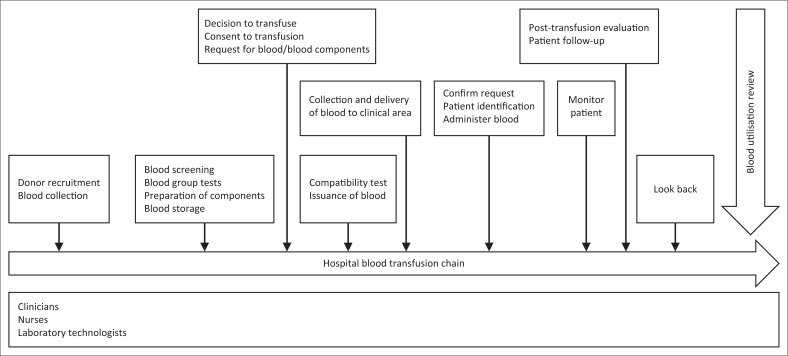
Blood transfusion chain.

The primary aim of this study was to assess the implementation of organisational management in the hospital blood transfusion services within Nairobi County, following WHO recommendations. The study further explored the level of implementation by private, faith-based and government hospitals and the role played by clinicians, nurses, laboratory technologists and managers (Figure 1).

## Methods

### Ethical considerations

Ethical clearance to carry out the study was sought from the Kenya Methodist University – Scientific and Ethics Review Committee (ethical approval number, HSM-3791-27115). Permission for data collection was also sought from individual hospital authorities. The hospital authorities gave verbal permission for their staff to participate. No patients’ data were used in the study. The individual respondents or hospital staff were required to complete an informed consent form prior to participation in the study.

### Study design

A cross-sectional descriptive study was conducted. Data were collected over 3 months, from June to August 2015. Questionnaires (Appendix 1) and first-hand observation guided by a checklist (Appendix 2) were used to collect the study data. The tools were developed and administered by the researcher. The hospitals selected were registered under the Kenya Medical Practitioners and Dentists Board or the Ministry of Health as the regulatory bodies and recognised by the Kenya National Blood Transfusion Service and Regional Society for Blood Transfusion Kenya. The selected facilities had a bed capacity of more than 20, so that they experienced sufficient demand for blood components to make installing a fully fledged quality management system in the hospital’s blood transfusion service cost-effective. Out of 25 hospitals in Nairobi County, 18 met the inclusion criteria.

The researcher visited each of the 18 health facilities selected and requested the participation of the institution. Permission was sought from the hospital authorities. An introduction letter was used when the administrator could not be reached. Once authorised, the researcher was introduced to one key staff member (manager) involved in blood transfusion services, who served as the contact person. A tour of the facility was provided, during which the self-administered questionnaires were issued to the respondents. A written informed consent form explaining the need, purpose, benefit and process of participation was filled out by the respondents before completing the questionnaires. The observation checklist was filled out by the researcher during the hospital visits.

Healthcare workers were the primary respondents. The respondents were selected through stratified random sampling and grouped into four categories: clinicians, nurses, medical laboratory technologists and managers involved in blood transfusion service. The respondents had at least 2 years of work experience in the blood transfusion service of the hospital. The inclusion of different cadres of staff captured the organisational management of the entire transfusion chain. The first respondent was the manager in charge of the blood transfusion service. The remaining respondents were selected randomly.

Overall organisational management was assessed by questionnaire items in four main areas: senior management team, hospital transfusion committee (HTC), quality officers and blood bank specialists. Senior management team (SMT) involvement was assessed via five specific yes-or-no questions. The respondents were asked if the SMT reviewed blood transfusion policies, ensured that staff held regular meetings, had appointed a quality officer, had appointed a blood transfusion consultant and reviewed audit reports.

Confirmation of the presence of a multidisciplinary HTC in each health facility was sought from the respective blood transfusion manager. The presence of a file for the HTC with minutes of meetings held was considered sufficient proof. Respondents were asked about the functionality and visibility of the committee within the hospital, and they indicated the performance of the committee for monitoring blood utilisation, development and promotion of the use of guidelines and carrying out audits of the processes within the service.

The quality officer and the blood transfusion specialists are key staff in blood transfusion service responsible for implementing the quality system and promoting best practices within the transfusion chain.^7,8^ Respondents were asked if these two designated staff were employed within their institutions.

### Statistical analyses

Data were coded and entered into a Microsoft 2013 Excel spreadsheet (Microsoft Corp., Redmond, Washington, United States). Data analysis was carried out using SPSS version 23 (IBM Corp., Armonk, New York, United States). Qualitative data were analysed using content and thematic analysis.

## Results

### Nairobi County hospital blood transfusion service

Of the 18 hospitals selected, 15 (83%) hospitals agreed to participate in the study. These hospitals were government, private and faith-based organisations, with five hospitals from each category. In total, 60 randomly selected health professionals from the 15 hospitals participated as respondents (100% response rate). A total of 33 (55%) respondents had worked in the blood transfusion services (BTS) for 4 years or fewer, and only two clinicians had served more than 5 years in their current station (Table 1).

**TABLE 1:** Profiles of the respondents, Nairobi County, Kenya, June–August 2015.

Variable	Number of years in hospital blood transfusion service			
	2–4	5–7	8–10	> 11
**Profession**				
Clinicians†	13	1	1	0
Nurses	7	4	2	2
Medical laboratory technologists	8	4	3	0
Managers‡	5	4	2	4
**Frequency**	33	13	8	6
**Percentage (%)**	55	21.7	13.3	10

† , Clinicians include clinical officers, medical officers, surgeons and physicians working in blood transfusion service.

‡ , Managers include administrative heads of blood transfusion services who could be medical lab technologists, nurses or clinicians by profession.

### Senior management team involvement in blood transfusion service

The overall performance of the senior management team was below average. A total of 27 respondents (45%) indicated that the senior management team met annually to review blood bank policies (Table 2). Having a protocol for staff meetings received the highest score (31 respondents, 52%), and review of audit reports received the lowest (14 respondents, 23.3%).

**TABLE 2:** Participants’ responses on extent of implementation of organisation management, in hospitals of Nairobi County, Kenya.

Variable	No. of respondents			
	Yes	%	No	%
**Senior management team activity†**				
Has a policy to ensure staff involved in blood transfusion service hold regular meetings	31	52.0	29	48.0
Meets annually to review BTS policies and strategies	27	45.0	33	55.0
Reviews BTS audit reports	14	23.3	46	76.7
**Hospital has transfusion committee**				
All hospitals	36	60.0	24	40.0
Government hospitals	20	33.3	40	66.7
Private hospitals	8	13.3	52	86.7
Faith-based hospitals	8	13.3	52	86.7
**Hospital transfusion committee activity**				
Monitors the use of blood components	22	36.7	38	63.3
Guidelines for BTS	22	36.7	38	63.3
Regular audit of the BTS	25	45.0	45	75.0
**Hospital has designated a blood transfusion consultant**	24	40.0	36	60.0
**Hospital has appointed a quality officer for the BTS**	12	20.0	48	80.0

BTS, blood transfusion services.

† , Senior management team includes: medical director, chief nursing officer, CEO, other patient services directors.

### Hospital transfusion committee

Hospital transfusion committees were present in nine participating hospitals (60%) (Table 2). All five government hospitals had established an HTC as opposed to only two private hospitals and two faith-based hospitals. The most commonly implemented roles of the HTCs were monitoring use of blood (22 respondents, 37%) and establishing guidelines (22 respondents, 37%). The least commonly implemented function of an HTC was audit of the service (15 respondents, 25%). This was further corroborated by the observation that only four (26.7%) of the hospitals had records that showed that audits were done.

### Quality officers

Twelve respondents (20%) reported that their hospital had a quality officer within the hospital blood transfusion service in Nairobi County (Table 2). Records of audit activities by the quality officer in the laboratory and the clinical area provided evidence that three (20%) of the hospitals had a designated quality officer.

### Blood transfusion specialists

Twenty-four health professionals (40%) indicated that their transfusion service had a clinician employed in the capacity of a blood transfusion specialist, who was responsible for providing expertise in therapeutic modalities and promoting best practice in transfusion medicine (Table 2). Records from the HTC meetings further indicated that 6 hospitals (40%) had a clearly designated staff member in this position.

Overall, based on how the respondents from each of the three types of hospital scored their BTS, organisation management implementation in BTS in Nairobi County was below average on the four areas assessed: SMT involvement (24 respondents, 40%), performance of the HTC (23 respondents, 38%), and appointment of blood transfusion specialists (24 respondents, 40%) and quality officers (12 respondents, 20%). Faith-based hospitals had the lowest level of implementation at 33.3%, private hospitals were at 42.5%, whereas government hospitals had the highest level of implementation at 60%. The performance of the government hospitals was boosted by close supervision and interaction with the RBTC.

## Discussion

Blood transfusion is a multistep, multidisciplinary service and involves different cadres of staff. This study was conducted in hospitals to explore the entire transfusion service chain. In this study more than half of the staff had served for less than 4 years in their current stations. Employee retention is important to create a pool of knowledgeable staff to serve in the HTC and as blood transfusion managers, quality officers or transfusion specialists. This study explored the implementation of organisational management as a foundational component of the quality system. The main pillar of this element was the involvement of senior management team in the blood transfusion service, which ensures the service obtains the necessary resources for appointment of key staff and supports other activities such as the establishment of an HTC. The senior management team scored poorly on the review of audit findings. This indicates that organisational management may not be able to incorporate audit findings to improve the service. Audits check blood wastage and monitor utilisation practices, blood storage and transfusion procedures. Failing to act on audit reports may lead to an inadequate or unsafe blood supply. Lack of tangible commitment from the hospital management is likely to cause poor implementation of recommendations in blood transfusion service.^9^

The establishment of HTCs in government hospitals was attributed to their closer interaction with the RBTC. In Kenya, there are six RBTCs; they are branches of the Kenya National Blood Transfusion Service and are mandated by the government to collect, process and distribute blood to all hospitals and provide oversight of transfusion services. This emphasises the role of the RBTC in providing administrative support to institutions to help them set up transfusion committees. Hospital blood transfusion committees have also been established in other countries, such as Brazil, where they are present in 63.4% of blood transfusion services.^10^ In a survey conducted in the Netherlands in 2012, all 76 hospitals were found to have HTCs.^11^

This study found that although the hospitals had established HTCs, they were not fully functional. Low performance on audits indicates that the hospitals may not implement measures to replenish blood bank stocks in good time. Audits may provide the HTC with information on blood bank stocks, wastage or other failures within the transfusion chain. Other studies have shown that without adequate support from senior management, resources and substantial authority, these committees cannot positively impact transfusion practice.^12^ According to the WHO, the role of an HTC is to ensure availability of blood components, audit transfusion practice, implement the national policy and monitor utilisation of blood in the hospital.^13^

The presence of a full-time quality officer in the blood transfusion service was very infrequent. This staff member is responsible for promoting adherence to best practices throughout the transfusion chain, implementing the HTC and senior management team decisions and ensuring that blood components are safe and services are adequate. The quality officer can be a nurse, a laboratory technologist or a clinician with sufficient experience in blood transfusion and quality management. Failure to appoint quality officers implies that problems that contribute towards blood shortage (such as wastage, misuse or adverse events) are not promptly captured, investigated and corrected through clearly defined guidelines. Although there have been mixed findings on employment of quality officers, with some developed countries also performing below average in this area, the vital role they play is not in question. In the United States, 42.9% of healthcare facilities had employed full-time quality assurance staff to investigate transfusion-related adverse reactions.^14^ In the 2012 Dutch national survey, all 76 hospitals sampled had appointed a quality officer to oversee transfusion safety.^11^

Less than half of the respondents indicated that they had a blood transfusion specialist in their institution. This specialist is a clinician with adequate experience and knowledge in blood transfusion who provides therapeutic advice, supports blood management and implements best practices, such as autologous transfusion and other strategies that eliminate, reduce or optimise blood transfusions.^15^ This shows that most of the hospitals in Nairobi do not have clearly defined guidelines to promote blood management and best practices in transfusion. Blood management is a multidisciplinary approach to improve patient clinical outcomes, while also reducing the need for allogeneic blood. This may include measures to prevent anaemia, such as iron supplementation to improve haemoglobin levels, and enhancement of coagulation function to limit bleeding in preoperative patients; other best practices include intraoperative blood recovery and use of fibrin sealants to reduce bleeding.^16^ These measures decrease and may eliminate the need for transfusion, thereby reducing wastage and inappropriate use of blood. A study conducted in England showed that 83% of hospitals had employed lead specialists for transfusion following the implementation of recommendations for ‘Better Blood Transfusion’.^9^

In general, Nairobi has not fully implemented the organisational management element despite WHO recommendations.^5^ The hospitals need strong involvement of senior management teams in transfusion service, effective transfusion committees and appointment of key staff (quality officers and transfusion specialists) in order to build a robust quality system. Through the quality system, the hospitals can then incorporate alternatives to donor blood, reduce wastage and maximise available blood components to benefit patients. The alternatives to allogeneic blood transfusion include autologous donations and intraoperative blood salvage.^16^ This would contribute significantly to safety, adequacy and effectiveness of the service. Robust blood transfusion services would significantly strengthen the healthcare system.

### Limitations

As this study was carried out in Nairobi County, the findings cannot be generalised to the entire country. Nairobi, as the capital city, has closer supervision from the RBTC and the Kenya National Blood Transfusion Service. The study involved healthcare organisations categorised as hospitals in the Kenya e-health database. Other institutions such as dispensaries or clinics offering blood transfusion services were not included.

### Recommendations

- The senior management teams should establish and empower HTCs to monitor utilisation and availability of blood components, audit transfusion practices and implement the national blood transfusion policy in the hospital.
- Hospital blood transfusion services should appoint key staff: quality officers and blood transfusion specialists. These two will maintain the quality system, promote transfusion best practices, investigate transfusion-related adverse reactions and support blood management practices, which eliminate, reduce and optimise blood transfusions.
- The RBTC should extend oversight and increase interaction with the private and faith-based hospitals to help establish strong HTCs in these facilities.
- Further research is needed to highlight the extent of implementation of the other WHO quality elements in Nairobi County.

### Conclusion

The SMTs in Nairobi county need to play a more visible role in strengthening the quality system in the blood transfusion service. The HTC, quality officers and blood transfusion specialists, where available, were not performing optimally. Hence, their roles in promoting transfusion best practices, blood management techniques to eliminate the need for transfusion and components preparation to maximise available units may not be realised. Essentially, Nairobi has not fully implemented the organisational management element to strengthen the blood transfusion service in its hospitals despite the WHO recommendation in 2002 for implementation of the quality management system.

### Trustworthiness

The data included in this study were collected and analysed by the authors. The results obtained and recommendations made are based on the information generated from the data collected. There were no updated or additional data added after the end of the data collection period.

### Reliability

Piloting of the questionnaire and observation checklist was done at the African Inland Church Kijabe Hospital. This is a faith-based hospital outside Nairobi County. This selection was made to avoid inadvertent sensitisation of the respondents who sometimes take up locum positions in neighbouring hospitals. The pre-test exercise and results were used to refine the research instruments.

### Validity

The study used both questionnaires and observation for triangulation of the results. The information provided by the respondents was verified by first-hand observation made by the researcher.

## Appendix 1

### Questionnaire

**GENERAL INFORMATION**

Respondent’s profession………………………………………………………………….

Years of service in this institution…………………………………… …………………

**INSTRUCTIONS**

→ Tick (√) the statement that best describe **your Hospital Blood Transfusion Service.**

→ Please cross-examine your blood transfusion service and provide accurate answers, which will contribute towards developing strategies to improve the blood transfusion service in Nairobi County.

**Section (a)**

1. Do you have a hospital transfusion committee?
  Yes ( ) No ( )
  If yes, answer question 2. **No – proceed** to question 3.
2. **Table:**
  Your Hospital Transfusion Committee has implemented the following:	Yes	No
  Guidelines for blood transfusion service		
  Measures to replenish blood bank stock		
  Regular audit of the blood transfusion service		
  Monitors the use of blood components by checking discard, expiry rate, C/T, TI, %T		
3. Does your department hold meetings to discuss the blood transfusion service?
  Yes ( ) No ( )
4. **Table:**
  The senior management team (medical director, chief nursing officer, CEO, other patient services directors)	Yes	No
  Meet annually to review the blood transfusion service policies and strategies		
  Has a policy to ensure staff involved in blood transfusion service hold regular meetings		
  Has appointed a quality officer to address blood transfusion issues		
  Has appointed a blood transfusion consultant who provides expertise in therapeutic modalities and promotes best practice in transfusion medicine		
  Reviews audit reports		
5. Is there evidence that the management reviews the laboratory (blood transfusion unit) internal quality control data?
  Yes ( ) No ( )
6. Is there a system to ensure traceability of blood components from donor to recipient and vice versa?
  Yes ( ) No ( )
7. **Table:**
  Blood/blood components	Strongly agree	Agree	Disagree	Strongly disagree
  Your hospital has capacity to prepare blood components (FFPs, platelets, packed red blood cells)				
8. **Table:**
  Blood components	Strongly agree	Agree	Disagree	Strongly disagree
  Your hospital has adequate storage capacity for FFPs				
  Your hospital has adequate storage capacity for Platelets				

**THANK YOU FOR PARTICIPATING**

## Appendix 2

### Observation Checklist

**Table:**

**For administrative use only** Serial number….……………………………………………..….. Type of facility………………………………..………………… Date: …………………………………………………………..		
**BLOOD TRANSFUSION SERVICE**	**Yes**	**No**
**Organization management**		
Hospital transfusion committee meeting minutes file		
Copy of approved agreement (contract) with blood supplier(s)		

**Table:**

**The hospital has capacity for preparation of:**		
Fresh frozen plasma		
Platelet concentrates		

**Table:**

**The blood transfusion unit has the following storage equipment**		
−30 degrees freezers for Fresh frozen plasma		
2–8 degrees fridges for packed cells		

**Table:**

**Agreement (contract) with supplier**		
Includes the means for maintaining blood inventory		
Includes a requirement for Notification in case a product is found to be seropositive		
Includes an **Alternative agreement to obtain blood from another institution in case of an emergency**		
Monitoring is done to ensure all deliveries meet stipulated requirements		

## References

[1] Mamaye Factsheet on Malawi’s blood services [homepage on the Internet]. 2015 [cited 2015 Sep]. Available from: www.mamaye.org/en/evidence/mamaye-factsheet-malawi’s-blood-services.

[2] World Health Organization Blood safety and availability [homepage on the Internet]. 2014 [Fact sheet, June 2014]. [Cited 2015 Sep] Available from: https://www.nation.co.ke/news/Kenya-faces-acute-blood-shortage/1056-2347170-iv22s5z/index.html.

[3] Kenya National Blood Transfusion Service KNBTS report: Kenya faces acute blood shortage [homepage on the Internet]. 2014 [cited 2015 Oct]. Available from: www.nation.co.ke ›News.

[4] Regional Society for Blood Transfusion Kenya (RSBTK) Blood collection report; 2013 Facility Haemovigilance Report. Nairobi: Regional Blood Transfusion Center.

[5] World Health Organization Quality systems for blood safety [homepage on the Internet]. 2002 [cited 2015 Nov]. Available from: www.who.int/bloodsafety/quality/en/AM_quality_system.pdf?ua=1.

[6] Ministry of Health, Pakistan National blood policy & strategic framework 2008–2012 for blood transfusion services in Pakistan [homepage on the Internet]. [Cited 2015 Sep]. Available from: www.nacp.gov.pk/…/National_Blood_policy_&_Strategicframework.

[7] Kenya National Blood Transfusion Service KNBTS About us [homepage on the Internet]. n.d. [cited 2015 Aug]. Available from: https://nbtskenya.or.ke/about-us/.

[8] Smith BR, Aguero-Rosenfeld M, Anastasi J, et al Educating medical students in laboratory medicine: A proposed curriculum. Am J Clin Pathol. 2010;133(4):533–542. 10.1309/AJCPQCT94SFERLNI 20231605

[9] Murphy MF, Howell C Survey of the implementation of the recommendations in the Health Service Circular 2002/009 ‘Better Blood Transfusion’. Transfus Med. 2005;15(6):453–460. 10.1111/j.1365-3148.2005.00621.x 16359415

[10] Carvalho RV, Brener S, Ferreira AM, Valle MC, Moraes-Souza H Transfusion practices committee of a public blood bank network in Minas Gerais, Brazil. Rev Bras Hematol Hemoter. 2012;34(6):416–420. 10.5581/1516-8484.20120104 23323064 PMC3545427

[11] Zijlker-Jansen PY, Janssen MP, Tilborgh-de Jong AJ, Schipperus MR, Wiersum-Osselton JC Quality indicators for the hospital transfusion chain: A national survey conducted in 100 Dutch hospitals. Vox Sang. 2015;109(3):287–295. 10.1111/vox.12281 25898854

[12] Liumbruno GM, Rafanelli D Appropriateness of blood transfusion and physicians’ education: A continuous challenge for hospital transfusion committees. Blood Transfus. 2012;10(1):1. 22044957 10.2450/2011.0056-11 PMC3258981

[13] World Health Organization Blood safety. Aide-Memoire for national blood programmes [homepage on the Internet]. 2003 [cited 2015 Sep]. Available from: http://www.who.int/bloodsafety/clinical_use/en/Aide-Memoire_23.3.04.pdf.

[14] Harvey AR, Basavaraju SV, Chung KW, Kuehnert MJ Transfusion-related adverse reactions reported to the National Healthcare Safety Network Hemovigilance Module, United States, 2010 to 2012. Transfusion. 2015;55(4):709–718. 10.1111/trf.12918 25371300 PMC7879051

[15] Tendulkar A Role of transfusion medicine consultant in peripheral blood stem cell transplant program [homepage on the Internet]. 2015 [cited 2015 Oct]. Available from: http://transmedcon2015.com/speakers/pdf/fourth_dec/hall-b/DR.%20ANITA%20A.%20TENDULKAR.pdf.

[16] American Association of Blood Banks (AABB) Technical manual. 17th ed. Bethesda, MA: AABB; 2011.

